# Cellular Prion Protein as a Novel Regulator of Vascular Plexus Stability During Retinal Angiogenesis

**DOI:** 10.1007/s11064-026-04867-8

**Published:** 2026-08-26

**Authors:** Bárbara Gomes da Rosa, Felipe Leser, Marco Aurélio Alves, Eliel de Souza Leite, Celina Garcia, Luiz Henrique Medeiros Geraldo, Rackele Ferreira do Amaral, Catarina Freitas, Flavia Regina Souza Lima

**Affiliations:** 1 Biomedical Sciences Institute, Universidade Federal do Rio de Janeiro, Rio de Janeiro, RJ Brazil; 2 Biomedical Sciences Institute, Universidade Federal do Rio de Janeiro, Macaé, RJ Brazil; 3 Department of Neurology, New York University, New York, NY USA; 4 School of Medicine, Yale University, New Haven, CT USA; 5 iBET, Instituto de Biologia Experimental e Tecnológica, Oeiras, Portugal; 6 Universidade Nova de Lisboa, Instituto de Tecnologia Química e Biológica António Xavier, Oeiras, Portugal

**Keywords:** Cellular prion protein, Angiogenesis, Retina, Neurovascular unit, Microglia, Laminin

## Abstract

Angiogenesis is essential not only during embryogenesis but also in adult physiology and disease, relying on vascular endothelial growth factor (VEGF) gradients to guide tip cell sprouting. Cellular prion protein (PrP^C^), expressed in several neurovascular unit (NVU) cell types, regulates neuronal and astrocytic differentiation through laminin binding, yet its role in angiogenesis remains understudied. Here, we investigated its function in retinal vascular development. In PrP^C^ knockout mice, the superficial retinal plexus exhibited increased tip cell density and branching, accompanied by microglial morphofunctional alterations and reduced *Vegf-c* expression, features consistent with impaired vascular stability. Conditioned media from PrP^C^ knockout microglia was sufficient to increase endothelial instability in vitro, indicating that altered microglial signalling can modulate vascular phenotype. Moreover, Laminin 411 / 511 isoforms and Collagen IV showed decreased expression in the retinal vascular basement membrane, supporting altered basement membrane composition. At later developmental stages, absence of PrP^C^ led to increased vertical sprouting into deeper retinal layers, resulting in transient hypervascularization of the deep plexus and defective laminin deposition across all vascular layers. These changes were accompanied by altered ZO-1 distribution and disrupted retinal layer organization, as indicated by reduced inter-plexus distance. Collectively, our findings identify PrP^C^ as regulator of sprouting angiogenesis and vascular stabilization in the developing retina. The data support a model in which PrP^C^ modulates neurovascular interactions, through both microglia-dependent and endothelial-intrinsic mechanisms, although the relative contribution of these components remains to be determined.

**Supplementary Information:**

The online version contains supplementary material available at https://doi.org/10.1007/s11064-026-04867-8.

## Introduction

Angiogenesis, the sprouting of new blood vessels from pre-existing ones, is the primary mechanism by which the vasculature in the developing central nervous system (CNS) expands and matures. In the CNS, the neural environment shapes vascular characteristics through coordinated interactions among endothelial cells, neural lineages and resident microglia [1]. This neurovascular interaction underlies the development of the blood-brain barrier (BBB) features, where junctional organization (e.g., through ZO-1) is determinant for proper barrier function [2, 3]. The mouse retina is an ideal model for CNS angiogenesis because its development is accessible and stereotyped [4]. At birth, the retina is largely avascular; astrocytes first migrate from the optic nerve head and establish a template for endothelial cells, which then sprout gradually to form the superficial plexus. Vertical sprouts subsequently project into the parenchyma and give rise to the deep and intermediate plexuses in a defined temporal sequence, followed by pruning and remodelling to refine the network [1, 5]. This process is driven by specialized endothelial tip cells that sense angiogenic gradients, notably VEGF, while adjacent stalk cells proliferate to extend the vessel. This tip-stalk balance is regulated by VEGF-Notch signalling: hypoxia-induced VEGF upregulates DLL4 expression, promoting tip cells, with high levels of VEGFR-2 and VEGFR-3 receptors and low levels of VEGFR-1 receptor [6–8]. Concurrently, lateral Notch signalling suppresses tip identity in adjacent stalk cells [6, 7, 9]. This complex crosstalk creates a balance between tip cells and stalk cells population, where [9] tip cells lead and guide migration while stalk cells proliferate expanding the vascular plexus [6, 10–12]. In the CNS, VEGF is secreted by astrocytes, neurons and microglia, promoting local control over sprouting and vessel stabilization [4, 9, 13, 14]. Microglia are closely associated with nascent vessels and sites of endothelial anastomosis, where they regulate sprouting, fusion and prunning responding to vessel-derived cues [15–17].

The prion cellular protein (PrP^C^) is highly expressed in human CNS, localized in outer cytoplasmic membrane, in rich cholesterol and sphingomyelin domains known as lipid rafts [18–20] where plays an important role on traffic and signalling of many molecules [21]. Despite being well characterized in prion diseases, its physiological role is not totally described. Due to the large number of ligands, PrP^C^ acts as a platform on cellular surface signalling molecules to physiological and behavioral events [22, 23]. Its expression in astrocytes is extremely important during CNS development, modulating neural survival and differentiation through its ligands, stress inducible protein 1 (STI1) and laminin [20, 24, 25].

PrP^C^ is also expressed in both mice and human endothelial cells [26]. In addition PrP^C^ absence on cerebral microvasculature endothelial cell membrane diminishes Occludin and Claudin-5 protein levels, which causes an increase of vascular permeability, suggesting that PrP^C^ is essential to endothelial monolayer integrity [27]. However, the field is lacking evidence for the role of PrP^C^ and its ligands in endothelial cells, particularly with regard to the regulation of angiogenesis during CNS development.

To investigate the role of PrP^C^ in microglial-vascular crosstalk, we analysed retinal angiogenesis in PrP^C^-deficient mice. We demonstrated that PrP^C^ deficiency is associated with increased but unstable vascular sprouting, accompanied by alterations in basement membrane composition and microglial behavior. In addition, conditioned media derived from PrP^C^-deficient microglia was sufficient to induce endothelial instability in vitro, supporting a contribution of microglia-derived signals to the observed phenotype. Together, this study supports a model in which PrP^C^ modulates vascular development through effects on neurovascular crosstalk. However, given the use of a global Prnp knockout model, the relative contribution of microglial versus endothelial cell-autonomous mechanisms cannot be fully resolved.

## Methods

### Animals

This study was approved by the Ethics Committee of the Center for Health Sciences (Centro de Ciências da Saúde—CCS) at the Federal University of Rio de Janeiro (UFRJ) (Protocol N. A6/19-001-16 and 060−24). All procedures strictly followed the “Guide for the Care and Use of Laboratory Animals” (published by the National Academy of Science, National Academy Press, Washington, D.C.). All efforts were made to minimize the number of animals used and their suffering.

For the in vivo experiments, neonatal Zrchl mice (postnatal day 0 to 21) were used in this work: PrP^C^ knockout animals (Prnp^0/0^) and their wild-type controls (Prnp^+/+^) [28]. In the lineage, the *Prnp* gene was deleted between amino acid residues 4 and 186 and replaced by a neomycin-resistance cassette. These animals were produced by continuous backcrossing of the initial 129/Sv × C57BL/6J mating. Both male and female mice were used in this study, and data were pooled for analysis.

### Retinal Dissection

Neonatal mice were euthanized by decapitation, whereas animals older than postnatal day 5 were anesthetized with isoflurane prior to sacrifice. The eyes were immediately enucleated by transecting the optic nerve. Eyeballs were immersion-fixed in 4% paraformaldehyde (PFA) in phosphate-buffered saline (PBS; 0.1 M, pH 7.4) for 5 min to preserve tissue integrity and facilitate dissection. Each globe was hemisected at the limbus (sclero-corneal junction), and the lens and vitreous body were carefully removed to expose the neural retina. Retinas were isolated by microdissection using fine forceps and microsurgical scissors. Dissected retinas were post-fixed in 4% PFA for 24 h at 4 °C to complete tissue fixation, following standardized protocols for whole-mount retinal preparation [29].

### Tissue Processing and Cryosectioning

For histological analysis, dissected retinas were rinsed in PBS and then cryoprotected by sequential immersion in 10%, 20% and 30% sucrose in PBS (overnight at 4 °C in each solution). Twenty-four hours prior to sectioning, retinas were embedded in Tissue-Tek^®^ O.C.T. Compound. A mold constructed from cryotubes was used: retinas were placed in the mold and completely submerged in O.C.T. The mold was then suspended in an isopropanol-cooled bath over liquid nitrogen to achieve controlled, progressive freezing. Cryosections (10 μm thickness) were cut on a cryostat at − 20 °C and collected onto glass slides pre-treated with 200 µg/mL Poly-L-lysine (Sigma P2636).

### Immunohistochemistry (Whole-Mount Retina)

Dissected and PFA-fixed retinas were washed in PBS and then permeabilized and blocked in 1% bovine serum albumin (BSA; Gibco) in PBS + 0.5% Triton X-100 for either overnight at 4 °C–2 h at room temperature. Samples were subsequently washed in Pblec buffer (1× PBS containing 1 mM CaCl₂, 1 mM MgCl₂, 0.1 mM MnCl₂, and 1% Triton X-100) and then incubated with primary antibodies anti-Laminin (1:200; Abcam; Cat.N^o^ ab7463), Anti-(IBA)−1 (1:200; Wako; Cat. N^o^ 019–197461), Anti-ZO-1 (1:300; Invitrogen; Cat. N^o^ 33–9100), Anti-Collagen IV (1:200; Abcam; Cat. N^o^ ab6586), Anti-GFAP (1:400; Dako; Cat. N^o^ Z033401), Anti-NG2 (1:200; Temecula; Cat. N^o^ Ab5320) and biotinylated isolectin B₄ (IB4, 1:200; Vector Laboratories; Cat.N^o^ B-1205) diluted in Pblec, overnight at 4 °C. For detection of IB4, samples were incubated with streptavidin conjugate in 0.5% BSA, 0.25% Triton X-100 in 1× PBS (pH 7.2) for 2 h at room temperature. Secondary antibody concentrations were used as follows: Alexa Fluor 568 (1:400; Molecular Probes), Alexa Fluor 488 (1:400; Molecular Probes); Alexa Fluor 546 (1:400; Molecular Probes); Alexa Fluor 647 (1:400; Molecular Probes). Eventually, tissues were stained with 4′,6-diamidino-2-phenylindole (DAPI; a nuclear marker). After PBS washes, retinas were mounted in Fluoromount Aqueous Mounting Medium (Sigma-Aldrich) and stored at 20 °C. Imaging was performed on a Leica TCS SP5/SP SPE confocal microscope; regions of interest were acquired with LAS X software.

### Quantitative Polymerase Chain Reaction (qPCR) Analysis

Total RNA was isolated from dissected retinas using TRIzol^®^ Extraction Reagent (Invitrogen) according to the manufacturer’s protocol. RNA concentration and purity were determined on a NanoDrop™ 2000/2000c spectrophotometer (Thermo Scientific™). cDNA was synthesized from total RNA using the High-Capacity cDNA Reverse Transcription Kit (Applied Biosystems). Resulting cDNA was amplified by quantitative PCR using SYBR^®^ Green Master Mix (Life Technologies, Cat. N^o^ 4367659) in a 10 µL reaction containing 4 µL cDNA (equivalent to 1,000 ng input RNA), 0.5 µL forward primer, 0.5 µL reverse primer (each at 0.5 µM final concentration), and 5 µL SYBR^®^ Green Master Mix. Thermal cycling and fluorescence detection were performed on a real-time PCR system. Relative gene expression was calculated using the 2^− ΔΔCT^ method, normalized to the endogenous control β-actin. The primer sequences used were as follows: *LAMα4*: forward: 5’- CAGCTGGACGACTACAACGC-3’ and reverse: 5’-ATGGTCGAGGGCCTGGTTA-3’; *LAMα5*: forward: 5’-CGGATGGGACCTGTGAAGA-3’ and reverse: 5’-GCTCTCCTGTGAAGTTCGGC-3’; *COL4α1*: forward: 5’- CTGGCACAAAAGGGACGAG-3’ and reverse: 5’- ACGTGGCCGAGAATTTCACC-3’; *CX3CR1*: forward: 5’ CTGTTATTTGGGCGACATTG-3’ and reverse: 5’ AACAGATTTCCCACCAGACC-3’: *ARG1*: forward: 5’ CTTGCGAGACGTAGACCCTG-3’ and reverse: 5’ TCCATCACCTTGCCAATCCC-3’: *VEGFA*: forward:5’-ATGAACTTTCTGCTGTCTTGG-3’ and reverse: 5’- ACCACTTCGTGATGATTCTGA-3’; *VEGFC*: forward:5’- TGATCCCCAGTCCGCGCGAG-3’ and reverse: 5’- CGGGCTCCGCTTCCGAGAAG-3’; *IL-4*: forward:5’-GAAGAACACCACAGAGAGTG-3’ and reverse:5’- AGCTCCATGAGAACACTAGA-3’; *IL1- β*: Forward:5’-TACAAGGAGAACCAAAGCAACGA-3’ and reverse:5’-TGCCGTCTTTCATTACACAGGA-3’: *β-actin*: forward:5’- TGGATCGGTTCCATCCTGG-3’ and reverse:5’-GCAGCTCAGTAACAGTCCGCCTAGC-3’.

### Quantitative Analysis of Angiogenesis, Microglia, and Astrocyte–Vessel Interactions

Confocal z-stacks of whole-mount retinas immunostained for IB4^+^ (endothelial marker) and IBA1^+^ (microglial marker) were analysed using ImageJ. *Tip cells* (IB4^+^ filopodia-bearing endothelial cells) and microglial cell bodies (IBA1^+^) were manually counted using the “Cell Counter” plugin. Vascular network metrics, including branching point density (ratio of vascular branch points per unit area), and vascular density (percentage of area occupied by vessels) were computed with *AngioTool* (v0.6a, National Cancer Institute) following published protocols [30, 31].

Collagen IV empty sleeves were identified as collagen IV-positive (collagen IV^+^), IB4-negative (IB4^−^) vascular structures and manually quantified in confocal images. To account for differences in vascular network size between samples, sleeve counts were normalized to vascular area measured from the corresponding image using AngioTolol software. For each field of view, the number of empty sleeves was divided by the vascular area, and normalized values were averaged to generate a single value per animal prior to statistical analysis. Both retinas from each animal were analysed, with eight fields of view acquired per animal. The biological replicate (n) corresponds to the individual animal.

Astrocyte–vessel association was quantified on 40x confocal images of retinas co-labeled for GFAP^+^ (astrocytes) and IB4^+^ (vessels). Colocalized GFAP/IB4 signal area was determined in *LAS X* software and normalized to the total vascular area measured by *AngioTool* [32, 33]. All quantitative data were plotted and statistically analysed in GraphPad Prism 8.0.

### Quantification of Laminin Deposition Relative to Vascular Area

Laminin immunofluorescence intensity was quantified using FIJI/ImageJ (NIH, USA). Briefly, IB4-stained images were first converted to 8-bit grayscale, and a threshold was applied using the Otsu method to generate a binary mask corresponding to IB4-positive (IB4⁺) vascular areas. Individual IB4⁺ regions were identified using the “Analyse Particles” function (minimum size: 10 px²) and saved as regions of interest (ROIs). The corresponding Laminin channel images were then used to measure fluorescence intensity exclusively within the IB4⁺ ROIs. Laminin signal was quantified as integrated density (IntDen), calculated as the product of the mean gray value and the number of pixels within each IB4⁺ selection. Final values were normalized to the total IB4⁺ area (px²) for each image, yielding a Laminin-to-IB4⁺ ratio that accounts for variability in vascular coverage across sections. All measurements were performed using a custom FIJI macro applied uniformly across all images.

### Microglial Morphological Analyses

Retinal microglial cells were identified by IBA1 immunostaining and analysed using a semi-automatic confocal image-processing pipeline to assess microglial morphometric parameters in ImageJ software (v1.54; National Institutes of Health, Bethesda, MD, USA), as previously described [34–36]. Briefly, z-stack confocal images of IBA1 immunostained retina whole-mounts from P0 and P5 *Prnp*⁰/⁰ and *Prnp*⁺/⁺ mice were converted to 8-bit, gray scale images. Unsharp Mask filter was applied to further improve microglial processes visualization and Despeckle step was performed to remove background single pixel noise. Images were then binarized and silhouette was used to calculate single cell area, perimeter and circularity index (4π[area]/[perimeter]^2^) using Analyse Particles function. Furthermore, cells were skeletonized and analysed using *Analyse Skeleton* plugin to quantify total, average and maximum branch length, number of branches, number of branch ramifications (junctions), number of triple and quadruple ramification points and number of endpoints. Finally, skeletonized microglial cells images were used to perform Sholl`s analysis, as previously described [36, 37] to calculate the number of intersections between the cell processes and concentric circles placed between the maximum radius of the cell soma and the radius surpassing the longest branch of the cell with an 1 μm step size. For morphology quantification, 100 cells were analysed per genotype, obtained from 3 animals from independent experiments.

### Human Brain Endothelial Cell Culture

Immortalized human brain microvascular endothelial cells (HBMECs; Nikolskaia et al., 2006) were kindly provided by Prof. Julio Scharfstein (Laboratory of Molecular and Cellular Immunology, Federal University of Rio de Janeiro). Cells were maintained in DMEM/F-12 (Gibco) supplemented with 10% fetal bovine serum (FBS; Gibco), 33 mM glucose, 2 mM L-glutamine, 15 mM sodium bicarbonate, 0.5 mg/mL penicillin–streptomycin, and 2.5 µg/mL Fungizone. Cultures were incubated at 37 °C in a humidified 5% CO₂ atmosphere until 80–90% confluence, then passaged using 0.025% trypsin-EDTA (Gibco). At each passage, aliquots were cryopreserved in FBS + 10% DMSO (Sigma) in liquid nitrogen, while the remainder were continued in culture.

### Primary Microglial Cell Culture

Primary microglial cultures were prepared from cerebral cortices of neonatal (P0–P2) ZrchI mice (*Prnp*^0/0^ and *Prnp*^+/+^) as described previously [38–40]. Meninges were carefully removed and cortical hemispheres dissected. Tissues were mechanically dissociated, then centrifuged and the cell pellet resuspended in DMEM/F-12 supplemented with 33 mM glucose, 2 mM L-glutamine, 15 mM sodium bicarbonate, 0.5 mg/mL penicillin–streptomycin, 2.5 µg/mL Fungizone, and 10% fetal bovine serum (FBS). Cells were plated in flasks pre-coated with 1.5 µg/mL poly-L-ornithine (Sigma) and incubated at 37 °C, 5% CO₂. After 14 days, confluent mixed glial cultures were shaken at 200 rpm to isolate microglia. The suspended microglia were collected and cultured at densities appropriate for each experiment.

### Preparation of Microglia-Conditioned Medium

Primary microglia were seeded at 1 × 10^6^ cells per well in 6-well plates (Techno Plastic Products, TPP) in DMEM/F-12 supplemented with 10% FBS. After attachment, cultures were washed with serum-free DMEM/F-12 to remove residual FBS. Cells were then maintained in serum-free DMEM/F-12 for 24 h at 37 °C, 5% CO_2_. Conditioned medium was collected, centrifuged and stored at − 20 °C for short-term use [39, 40].

### Endothelial Tube Formation Assay (Matrigel)

HBMECs were seeded at 3 × 10^4^ cells per well (0.2 cm²) onto Matrigel (BD Biosciences). Cells were cultured for 24 h at 37 °C, 5% CO₂ in microglia-conditioned medium (*Prnp*^+/+^ or *Prnp*^0/0^) or serum-free DMEM/F-12 (negative control). To ensure equal viable cell seeding on Matrigel, samples of each cell suspension were mixed with 0.4% trypan blue and counted in a Neubauer chamber. Cells were plated in triplicate. Tubulogenesis was monitored by phase-contrast microscopy (Nikon TE300) at 2, 4, 6, 8, 24, and 48 h to determine the optimal assay time based on sprout formation, which results in the network morphology characteristic of angiogenic differentiation and in the surface occupied by endothelial cells. Images acquired at 24 h and 48 h were analysed in ImageJ. Endothelial-occupied area was measured using the “Freehand Selections” tool and branching points were counted with the “Cell Counter” plugin. Quantitative results were plotted in GraphPad Prism 8.0.

### Scratch Wound Healing Assay

Cell migration was assessed using a scratch wound assays as previously described [39, 40]. HBMECs were seeded at 2.5 × 10^5^ cells per well in 24-well plates (Techno Plastic Products, TPP). After 6 h to allow cell adhesion, Cultures were treated with 10 µM cytosine-β-D-arabinofuranoside (Ara-C; Sigma; Cat. No. C1768) overnight to minimize cell proliferation. A linear “scratch” wound was created in the confluent monolayer using a sterile pipette tip. Wells were washed with serum-free DMEM/F-12 to remove detached cells. Cells were then incubated with microglia-conditioned medium for 24 h at 37 °C, 5% CO₂. Images of the wound area were captured immediately after scratching (0 h) and at 24 h using an inverted Nikon Eclipse microscope. Wound closure was quantified using ImageJ by measuring the cell-occupied area within the scratch. Data were plotted in GraphPad Prism 8.0.

### Imaging Quantification and Biological Replicates

Biological replicates (*n*) refer to independent animals. For imaging-based analyses, multiple fields of view were acquired per retina and quantified individually and averaged per animal prior to statistical analysis.

For each animal, both retinas were analysed. Each retina was subdivided into four radial regions, and one field of view was acquired per region, resulting in eight fields of view per animal.

Values from all fields of view were averaged across both retinas to generate a single measurement per animal. Statistical analyses were performed using these per-animal means, ensuring that the biological replicate corresponds to the individual animal rather than multiple measurements from the same specimen, thereby avoiding pseudo replication.

Animals used in these analyses represent independent biological samples. Although animals from the same litter were occasionally included, replicates were drawn from multiple litters and processed in parallel using identical experimental protocols and imaging acquisition settings to minimize batch effects.

### Statistical Analysis

All data were analysed using GraphPad Prism 8.0. Comparisons between two groups were performed using an unpaired Student’s t test. For comparisons among three or more groups, one way ANOVA followed by Tukey’s post hoc test was used. A 95% confidence interval was applied, and p values less than 0.05 were considered statistically significant. Significance is denoted as follows: p less than 0.05, p less than 0.01, p less than 0.001, and p less than 0.0001.

Effect sizes were calculated using Cohen’s d, defined as the standardized mean difference between groups using the pooled standard deviation. For each primary phenotypic comparison, 95% confidence intervals for Cohen’s d were estimated using a standard large sample approximation. Calculations were performed using GraphPad Prism 8.0. In addition, approximate sample sizes required to achieve 80% statistical power at alpha 0.05, two tailed, were calculated using the observed effect sizes as planning estimates for future studies. These analyses are reported in Supplementary Fig. 2.

## Results

### Prnp^0/0^ Vascular Plexus Exhibits Increased Sprouting and Branching During Retinal Development

**Fig. 1:**
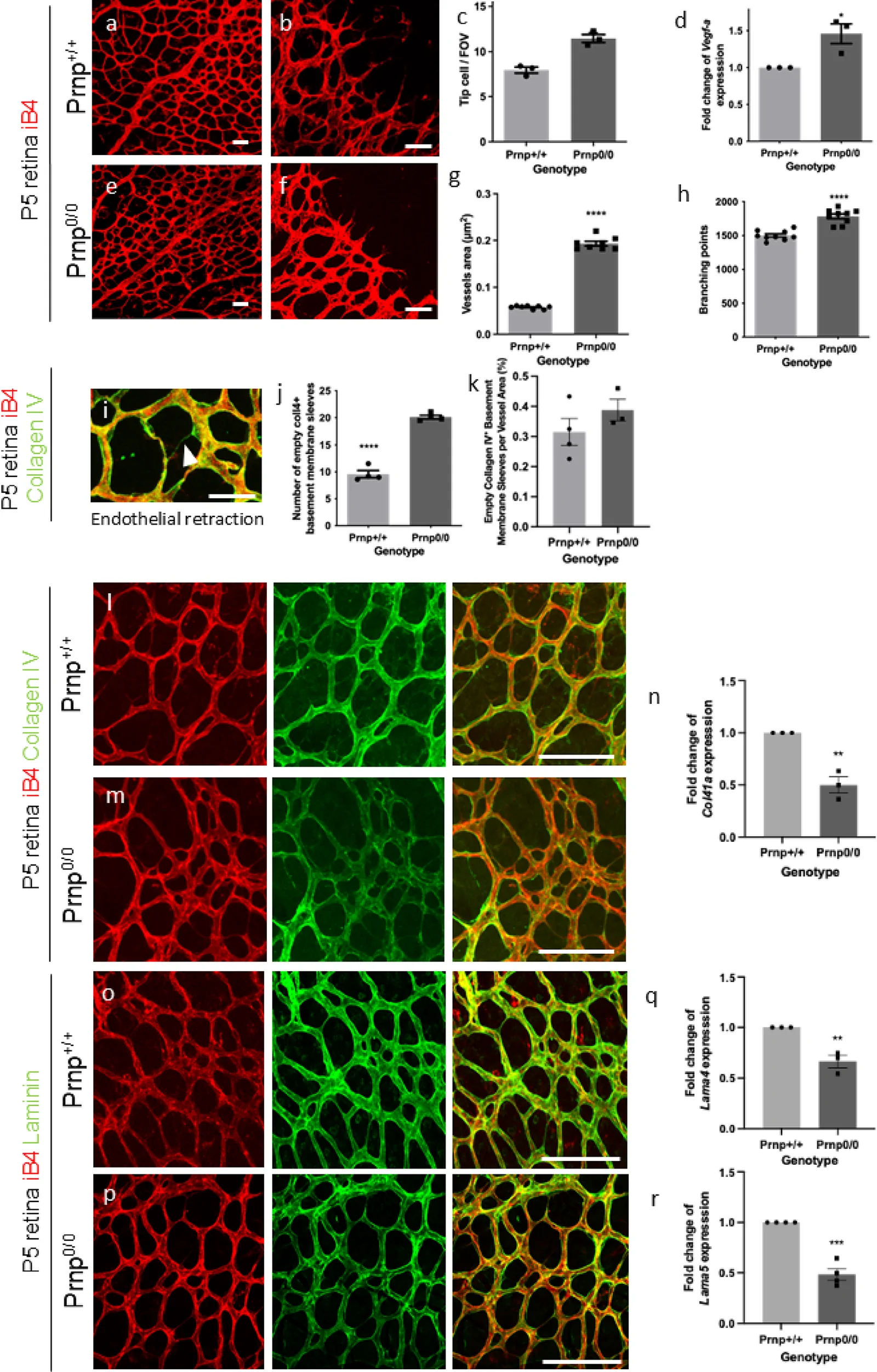
PrP^C^ regulated angiogenic sprouting and vascular stability in the superficial retinal vascular plexus. Confocal images of P5 mice retinas illustrating the effect of *Prnp* deletion on retinal angiogenesis. **a**,** b** Isolectin B4 (IB4) staining (red) of the superficial vascular plexus in *Prnp*^+/+^ and *Prnp*^0/0^ mice; **c** note increased number of tip cells in *Prnp*^0/0^ retinas compared to the *Prnp*^+/+^ retinas. **d** Quantification of *Vegf-a* expression (qPCR analysis) data is normalized for *β-actin* and expressed as fold change in respect to *Prnp*^+/+^. **e**,** f** Representative images of the vascular network. **g** Quantification of vascular area. **h** Quantification of branching points. **i** Double immunostaining for IB4 (red; endothelial cells) and Collagen IV (green; vascular basement membrane) revealed collagen IV⁺ basement membrane sleeves (empty sleeves), defined as Collagen IV-^+^ structures lacking endothelial cells, indicative of vascular regression. **j** Quantification of the number of empty collagen IV⁺ basement membrane sleeves in Prnp⁺/⁺ and Prnp⁰/⁰ retinas. **k** Quantification of empty collagen IV⁺ basement membrane sleeves normalized to vessel area to account for differences in vascular density between genotypes. Images were acquired using a 20× objective. Scale bar = 50 μm. Data are presented as mean ± SEM. For tip cell and *Vegf-a* analyses, *n* = 3 animals per genotype; for vascular density and branching analyses, *n* = 9 animals per genotype. The two types of symbols correspond to the genotypes (circles to *Prnp*^+/+^ and squares to *Prnp*^0/0^). Statistical analysis was performed using an unpaired two-tailed Student’s t-test. Data are presented as mean ± SEM. *p < 0.05; ****p < 0.0001. For studies related to Laminin and Collagen IV expression in the retinal vascular basement membrane, immunohistochemical and gene expression analyses were performed to evaluate extracellular matrix components in the retinal vasculature of P5 mice. **l**,** m** Representative confocal images of retinas stained for IB4 (red) and Collagen IV (green) showing reduced Collagen IV deposition in *Prnp*^0/0^ mice. **n** qPCR analysis for *Col4α1* expression from *Prnp*^+/+^ and *Prnp*^0/0^ retinas. **o**,** p** Representative confocal images of retinas from *Prnp*^+/+^ and *Prnp*^0/0^ mice stained with IB4 (red) and Laminin (green). **q**,** r** qPCR analysis of retinal tissue for laminin isoforms *Lama411* (laminin α4) and *Lama511* (laminin α5) expression. Images were acquired using a 63× objective. A total of *n* = 3 animals per genotype were analysed. Scale bar = 50 μm. Statistical analysis was performed using an unpaired two-tailed Student’s t-test. Data are presented as mean ± SEM. **p < 0.01; ***p < 0.001

To investigate the role of PrP^C^ in angiogenesis during CNS development, we analysed retinas from PrP^C^ knockout mice (Prnp^0/0^) at postnatal day 5 (P5). No overt differences in body or eye size were observed between the two genotypes during the developmental stages analysed. Immunofluorescence revealed an approximately 44% increase in IB4^+^ tip cells at the expanding vascular front in Prnp^0/0^ retinas compared to controls (Prnp^+/+^) (Fig. 1a–c). Consistent with enhanced sprouting activity, Prnp^0/0^ retinas also displayed a significant upregulation of Vegf-a mRNA relative to Prnp^+/+^ retinas (Fig. 1d). Morphometric analysis of the P5 vascular plexus showed that Prnp^0/0^ retinas exhibited a threefold increase in vascular density and an 18% increase in branching points compared to controls (Fig. 1e-h).

To assess vessel maturation and remodelling, we performed double labeling for IB4 and Collagen IV to visualize endothelial tubular structures (IB4^+^) and the surrounding basement membrane (Collagen IV^+^). In intact and maturing vascular segments, Collagen IV encased IB4^+^ endothelial tubes, whereas regression events appeared as Collagen IV^+^ but IB4^-^ segments, referred to as empty sleeves (Fig. 1i, j, k). These structures represent remnant basal lamina following endothelial retraction and provide an indirect readout of vessel regression. Quantification revealed that Prnp^0/0^ retinas exhibited an increased absolute number of empty sleeves (Fig. 1j). However, after normalization to vascular area, this difference was no longer statistically significant (Fig. 1k).

### Prnp^0/0^ Retina Exhibited a Reduction in the Expression Levels of Collagen-IV and Laminin

To investigate whether vascular basement membrane components were altered in Prnp^0/0^ retinas, we assessed the expression of collagen IV and laminin, two major constituents of the vascular extracellular matrix. At P5, double immunofluorescence for IB4 and Collagen IV revealed reduced Collagen IV labelling in Prnp^0/0^ retinas, consistent with an approximately 50% decrease in Col4α1 mRNA levels compared to Prnp^+/+^ controls (Fig. 1l–n). We next examined the laminin α4 and α5 chains, which are components of the Laminin-411 and Laminin-511 isoforms that predominate in the postnatal retinal vasculature [41]. mRNA levels for both laminin chains were reduced by approximately 50% in Prnp^0/0^ retinas relative to controls (Fig. 1q, r). Immunofluorescence analysis likewise indicated reduced laminin labelling around vascular structures (Fig. 1o, p, p). Collectively, these findings demonstrate that loss of PrP^C^ is associated with reduced expression of key vascular basement membrane components in the developing retina.

### P5 Prnp^0/0^ Retina Presented Enhanced Microglial Density in Vascular Front with Morphofunctional Changes

**Fig. 2:**
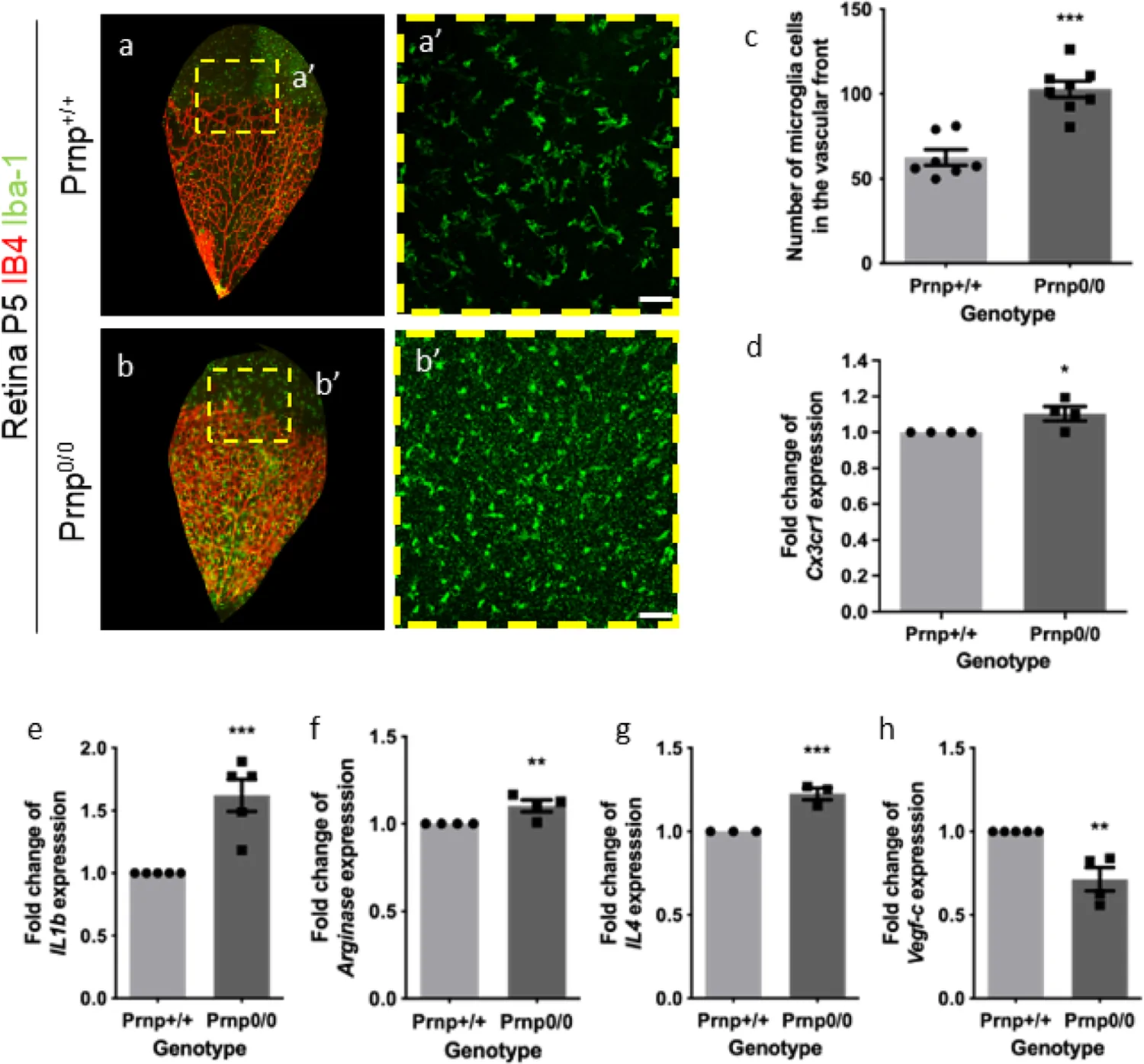
Absence of PrP^C^ altered microglial density and gene expression at the retinal vascular front. Retinas from P5 *Prnp*⁺/⁺ and *Prnp*⁰/⁰ mice were analysed to evaluate microglial distribution and phenotype during retinal vascular development. **a**,** b** Representative confocal images of whole-mount retinas stained with isolectin B4 (IB4, red) to label endothelial cells and IBA-1 (green) to label microglia. The boxed regions indicate the vascular front. **a′**,** b′** Higher magnification images of the vascular front showing an increased number of IBA-1^+^ microglial cells in *Prnp*⁰/⁰ retinas compared with *Prnp*⁺/⁺ controls. **c** Quantification of microglial cells at the vascular front. **d–h** Gene expression analyses by qPCR for *Cx3cr1* (microglial receptor), pro-inflammatory (*Il1β*) and anti-inflammatory (*Arg1* and *Il4*) markers and *Vegf-c* in retinas. Images **a**,** b** were acquired using a 20× objective (scale bar = 50 μm). Data are presented as mean ± SEM. *n* = 7 animals per genotype for microglial quantification (**c**); *n* = 4 or 5 animals per genotype for *Cx3cr1*, *Il1β*, *Arg1* and *Vegf-c* expression (**d–h**); *n* = 3 animals per genotype for *Il4* expression (**g**)

Considering the marked increase in tip cell number in *Prnp*^0/0^ retinas and the established role of microglia in guiding sprouting, branching and anastomosis of the expanding vascular plexus [42] we examined microglial distribution at the vascular front. Immunostaining for IBA-1 revealed a 64% increase in microglial density in *Prnp*^0/0^ retinas compared to *Prnp*^+/+^ controls (Fig. 2a–c). To determine if this was associated with altered neurovascular signalling, we examined VEGF-C, a key microglial regulator of vascular sprouting and stabilization [16]. *Vegf-c* mRNA levels were significantly reduced in *Prnp*^0/0^ retinas, suggesting reduced pro-stabilizing signals in the absence of PrP^C^ (Fig. 2h). We note that this measurement reflects whole-retina expression and therefore cannot be attributed to a specific cellular source.

To further explore mechanisms associated with microglial distribution, we examined the CX3CR1 fractalkine receptor, a regulator of microglial migration and neurovascular interactions. We observed a significant upregulation of *Cx3cr1* mRNA, which together with reduced *Vegf-c* is consistent with altered signalling relevant to vascular remodeling (Fig. 2d, h). This altered state was further supported by changes in genes commonly associated with inflammatory and remodelling responses. Whole-retina analysis revealed increased *Il1b* mRNA, together with elevated expression of *Arg1* and *Il4* (Fig. 2e - g). Because these measurements reflect contributions from multiple retinal cell types, they should be interpreted as a composite transcriptional profile rather than attributed to a specific population.

**Fig. 3:**
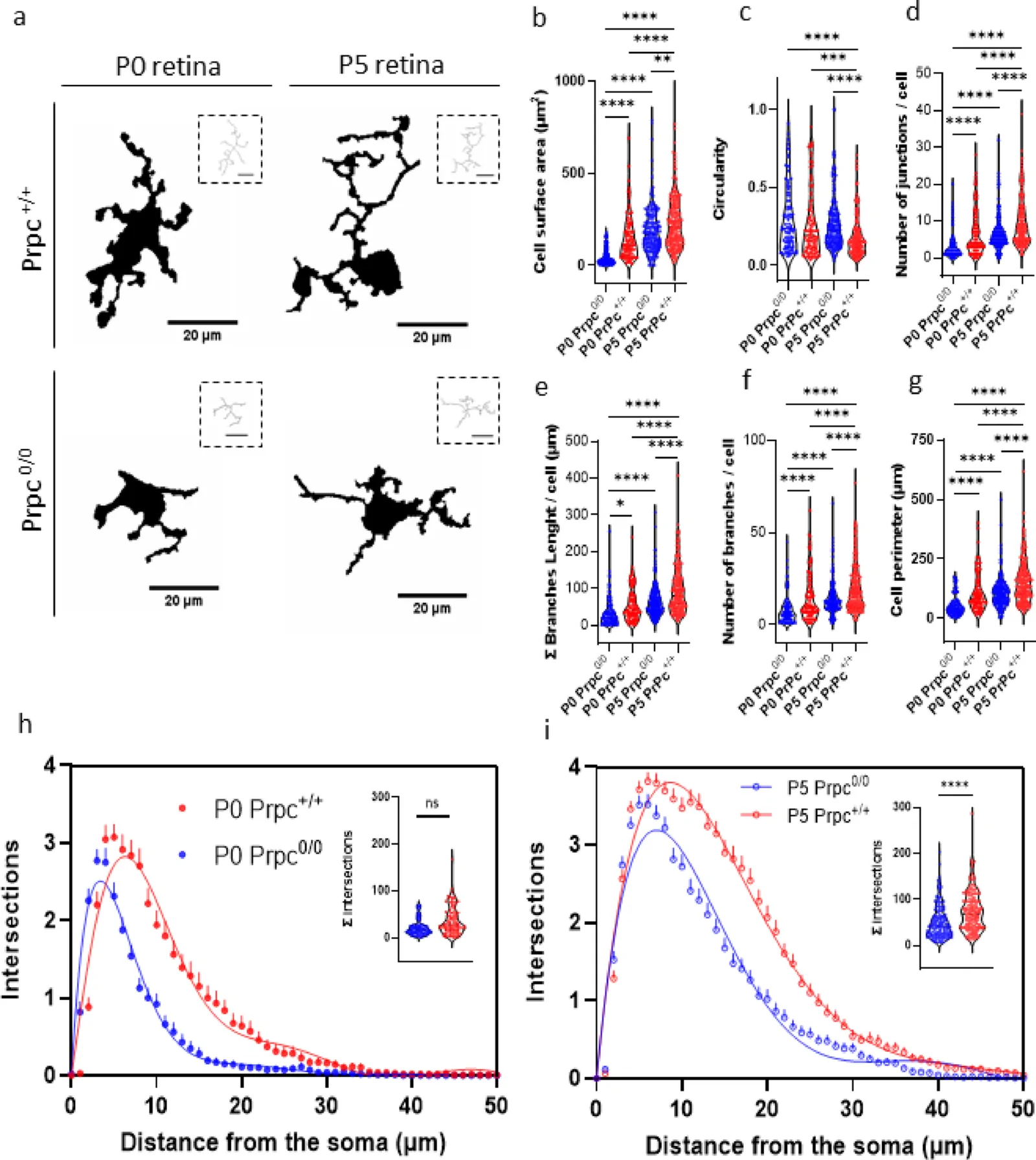
Absence of PrP^C^ altered microglial morphological differentiation in the neonatal retina. **a** Representative images of IBA1 stained microglial cells from P0 and P5 mouse retinas of *Prnp*⁺/⁺ and *Prnp*⁰/⁰ genotypes. Dashed boxes show representative examples of the analysed skeletonized cell structure. Scale bar = 20 μm. **b–g** Quantification of microglial morphometric parameters including cell surface area (µm^2^) (**b**), circularity (**c**), number of junctions per cell (**d**), total branch length per cell (µm) (**e**), number of branches per cell (**f**) and cell perimeter (µm) (**g**) comparing microglial cells from *Prnp*⁺/⁺ and *Prnp*⁰/⁰ retinas at P0 and P5. Ordinary one-way ANOVA with Tukey’s multiple comparisons test. **h**,** i** Sholl analysis of skeletonized microglial cells at P0 (**h**) and P5 (**i**), showing the number of intersections of microglial processes with concentric circles at increasing distances from the soma (1 μm step size). Curves represent mean ± SEM fitted with a polynomial regression. Insets show the total number of intersections across all distances from the soma for each experimental group. Mann Whitney Test. *p < 0.05; **p < 0.01; ***p < 0.001; ****p < 0.0001. One hundred cells were analysed per genotype, obtained from 3 animals from independent experiments

To determine whether these transcriptional alterations were accompanied by structural changes in microglia morphology, we next performed morphometric analysis of *Prnp*⁺/⁺ and *Prnp*⁰/⁰ at P0 and P5, two developmental stages that bracket the window of superficial retinal vascularization. At P0 microglia is present but the retina is essentially avascular, whereas P5 corresponds to the period of superficial plexus expansion, with microglia actively regulating tip cell sprouting, anastomosis and stabilization. At P0, *Prnp*⁰/⁰ microglia already displayed an immature phenotype, characterized by reduced cell surface area and decrease in morphological complexity, including fewer and shorter processes, reduced branching, smaller perimeter and lower Sholl intersection counts compared to *Prnp*⁺/⁺ (Fig. 3). *Prnp*⁰/⁰ cells also exhibited a higher proportion of amoeboid-like morphology, as indicated by increased circularity, consistent with a more reactive state (Fig. 3c). Although both genotypes exhibited a developmental increase in microglia size and complexity by P5, *Prnp*⁰/⁰ microglia remained consistently less complex across all parameters. At P5, they retained shorter and fewer processes, fewer branch points, reduced branch length, and a markedly smaller perimeter than *Prnp*^+/+^ controls (Fig. 3a–i). The proportion of circular, ameboid-like cells also remained higher in P5 *Prnp*⁰/⁰ retinas, further indicating a persistent reactive phenotype (Fig. 3c). *Prnp*⁰/⁰ microglia did not acquire the typical morphology of maturing homeostatic microglia. Together these results indicate that *Prnp*⁰/⁰ retinas exhibit increased microglial abundance accompanied by altered transcriptional and morphological features during early retinal vascular development.

As other neurovascular cellular components contribute to retinal angiogenesis, we next investigated whether endothelial-astrocyte or endothelial-pericyte interactions were affected in P5 *Prnp*^0/0^ retinas. We performed double-immunostaining for GFAP and IB4 which revealed an intact astrocytic scaffold and normal vessel ensheathing, with no significant changes in *Gfap* mRNA levels in the retina *Prnp*^0/0^ retinas (Supplementary Fig. 1a - d). Similarly, as pericytes are part of the neurovascular unit and critical for vessel stability, we assessed pericyte recruitment using the NG2 marker. Double immunolabelling with IB4 and NG2 revealed that there were no significant differences in the spatial organization of pericytes, fluorescence intensity or pericyte coverage between *Prnp*⁰/⁰ and *Prnp*^+/+^ retinas (Supplementary Fig. 1e - h). These results suggest that the vascular phenotype observed in *Prnp*⁰/⁰ retinas is not associated with overt alterations in astrocyte- or pericyte-endothelium organization. Effect size estimates for the primary phenotypic comparisons are provided in Supplementary Fig. 2.

### Endothelial Tubular Networks Treated with Prnp^0/0^ Microglia-Conditioned Medium Showed Increased Instability and Migration In Vitro

**Fig. 4:**
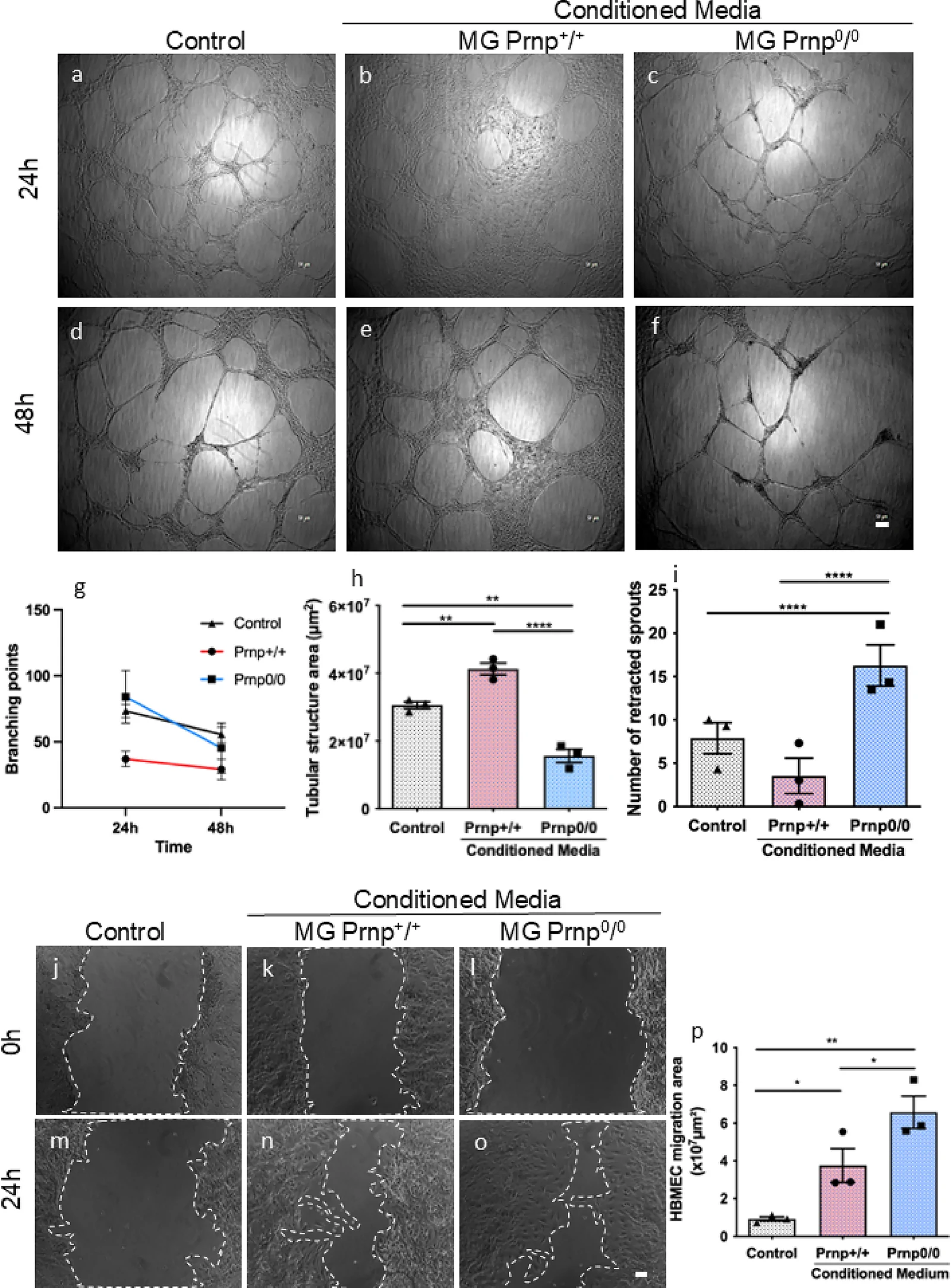
PrP^C^ modulated the microglial secretome, affecting endothelial tube stability and migration in vitro. HBMECs were cultured on Matrigel and treated with conditioned medium (CM) derived from microglia expressing PrP^C^ (MG *Prnp*^+/+)^ or lacking PrP^C^ (MG *Prnp*^0/0^). **a–c** Representative images of tubular structures formed after 24 h under control conditions and in the presence of CM from MG *Prnp*^+/+^ or MG *Prnp*^0/0^. **d–f** Representative images of the same conditions after 48 h of culture. **g** Quantification of branching points over time of cultures treated with CM from MG *Prnp*^0/0^ and MG *Prnp*^+/+^. **h** Quantification of tubular structures of cultures treated with CM from MG *Prnp*^0/0^ and MG *Prnp*^+/+^ after 48 h. **i** Quantification of retracted sprout fusion sites demonstrated that tubular structures formed in the presence of MG *Prnp*^0/0^ CM were significantly more unstable than those formed under the other conditions after 48 h. To evaluate endothelial migration, HBMECs were subjected to a scratch wound assay and treated with CM from MG *Prnp*^+/+^ or MG *Prnp*^0/0^. **j–l** Representative images of wound areas immediately after scratching (0 h) under control conditions and following treatment with MG *Prnp*^+/+^ or MG *Prnp*^0/0^ CM (**m**–**o**). Representative images of the same fields after 24 h. **p** Quantification of scratch wound assay. Images were acquired using 4× (A–F) or 10× (J–O) objectives. Scale bar = 50 μm. Data represent mean ± SEM from three independent experiments performed in triplicate (*n* = 3). Statistical analysis was performed using one-way ANOVA followed by Tukey’s multiple comparison test. *p < 0.05; **p < 0.01; ***p < 0.001; ****p < 0.0001

To investigate whether microglial secreted factors can modulate endothelial behavior, we examined the influence of microglial conditioned media (CM) on endothelial tube formation. HBMECs were cultured in Matrigel and treated with CM from primary microglial cell cultures from *Prnp*^0/0^ (Prnp^0/0^ MG) and *Prnp*^+/+^ (*Prnp*^+/+^ MG) mice (Fig. 4), using serum-free DMEM/F-12 as a control (Fig. 4a, d). After 24 h, we observed tubular structures across all conditions (Fig. 4a–c), with *Prnp*^0/0^ MG-derived CM condition associated with a significant increase in branching points (Fig. 4c, g), but a reduced total network area (Fig. 4h). These findings indicate that, although *Prnp*^0/0^-derived factors enhanced initial sprouting, they do not sustain vessel network expansion, consistent with reduced vessel stability. To follow the progression of the tubular plexus, we imaged the same regions at 48 h. We observed endothelial retraction in all conditions tested, as indicated by decreased branching points overtime (Fig. 4d–g). This decrease was more pronounced in in *Prnp*^0/0^ MG-derived CM (Fig. 4f, g), indicating higher instability (Fig. 4i). Moreover, a higher proportion of connected sprouts underwent regression in *Prnp*^0/0^ MG CM condition. Together, these results indicate that microglia-derived factors from Prnp^0/0^ retinas are sufficient to promote increased branching and reduced stability of endothelial networks in vitro.

We next investigated the influence of the Prnp^0/0^ microglial secretome on endothelial migration, a hallmark of the tip cell phenotype. To ensure that wound closure reflected cell migration rather than proliferation, HBMEC cultures were pretreated with cytosine-β-D-arabinofuranoside (Ara-C) prior to scratch induction. Using this assay, we found that Prnp^0/0^ MG-derived CM significantly increased HBMEC migration, as evidenced by a 75.2% increase in wound closure area after 24 h (Fig. 4l, o, p) compared to Prnp^+/+^ MG-derived CM (Fig. 4k, n, p). Collectively, these data suggest that the microglial secretome modulates endothelial cell migration and may influence tip and stalk cell dynamics in vitro.

### Expansion and Remodelling of the Superficial Vascular Plexus were Altered in Prnp^0/0^ Retinas

**Fig. 5:**
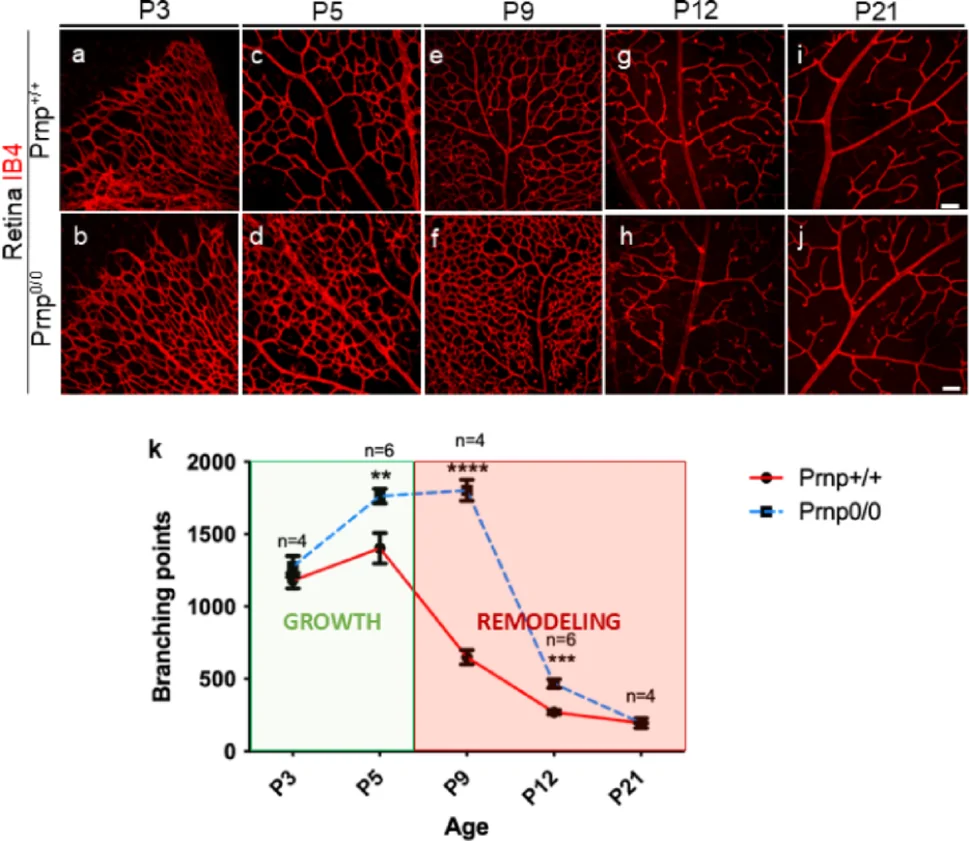
PrP^C^ regulated vascular density and remodelling in the superficial retinal vascular plexus during postnatal development. Confocal images of mouse retinas at postnatal days P3, P5, P9, P12 and P21 stained with IB4 (red) to visualize the superficial vascular plexus. **a–j** Representative images from *Prnp*⁺/⁺ and *Prnp*⁰/⁰ mice at the indicated developmental stages. **k** Quantification of branching points across developmental stages. While *Prnp*⁺/⁺ retinas begun the remodelling phase between P5 and P9, *Prnp*⁰/**⁰** retinas exhibited a delayed remodelling process occurring predominantly between P9 and P12. Images were acquired using a 20× objective. Scale bar = 50 μm. Data are presented as mean ± SEM. *n* = 4–6 animals per genotype depending on the developmental stage. Statistical significance was determined using one-way ANOVA with Tukey’s multiple comparison test. **p < 0.01; ***p < 0.001

**Fig. 6:**
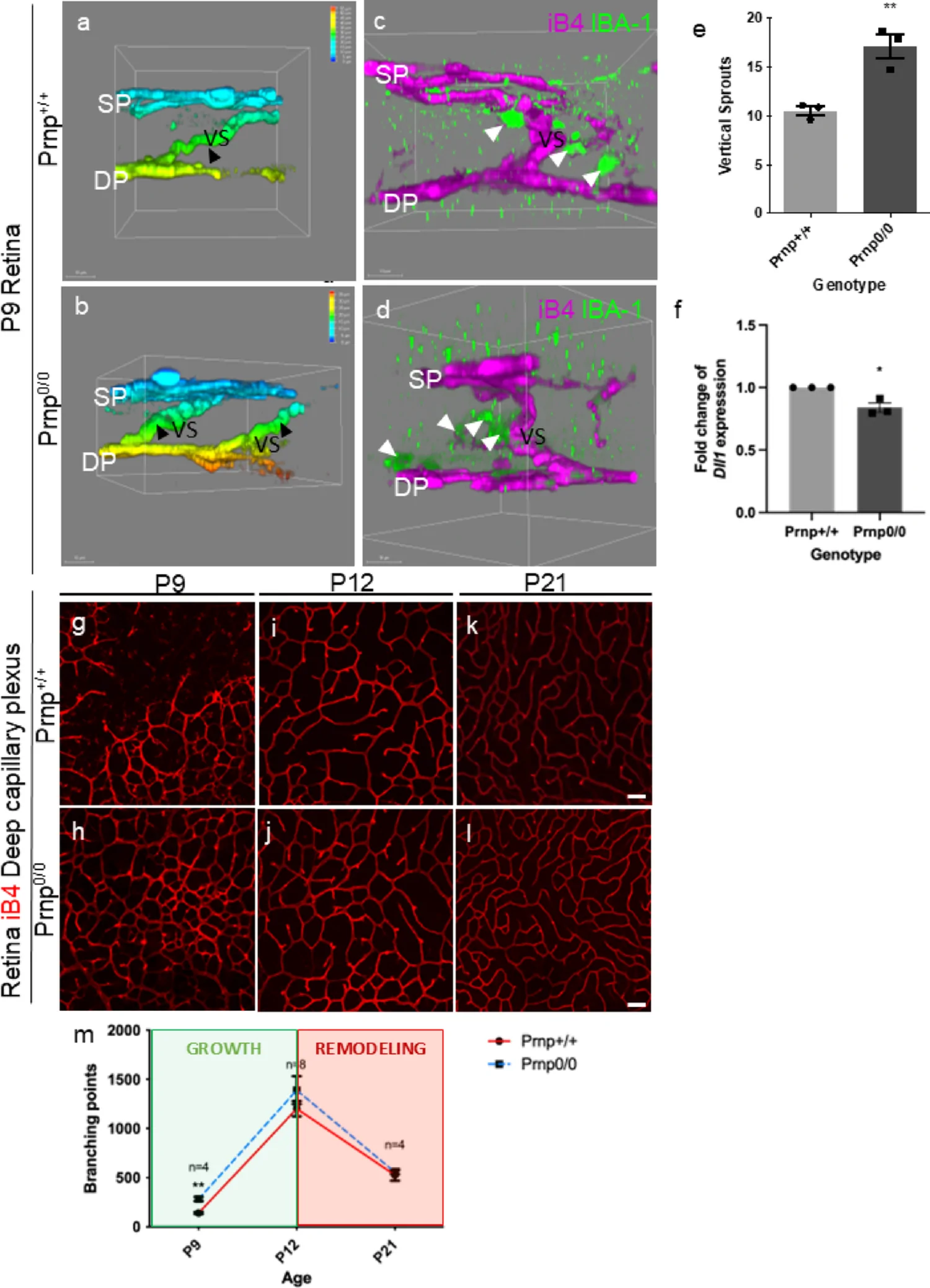
PrP^C^ regulated vertical sprouting and formation of the deep retinal vascular plexus during postnatal development. Three-dimensional confocal analysis of retinal vasculature was performed to evaluate vertical sprouting and formation of the deep vascular plexus in *Prnp*⁺/⁺ and *Prnp*⁰/⁰ mice. **a**,** b** 3D reconstruction of P9 retinas stained with IB4 showing the superficial plexus (SP), deep plexus (DP), and vertical sprouts (VS) connecting both vascular layers. *Prnp*⁰/⁰ retinas exhibited an increased number of vertical sprouts compared with *Prnp*⁺/⁺ animals. **c**,** d** Confocal images of P9 retinas stained for IB4 (magenta) and IBA-1 (green) revealed vertical sprouts extending between the superficial and deep vascular layers. These sprouts are closely associated with IBA-1^+^ microglial cells (arrowheads), indicating a spatial interaction between microglia and endothelial sprouts. **e** Quantification of vertical sprouts. **f** qPCR analysis for *Dll1* expression. To evaluate formation of the deep vascular plexus, retinas from different developmental stages were analysed. **g**,** i**,** k** Representative images of the deep capillary plexus in *Prnp*⁺/⁺ mice at P9, P12, and P21, respectively. **h**,** j**,** l** Corresponding images from *Prnp*⁰/⁰ mice at the same developmental stages. **m** Quantification of branching points in the deep plexus at P9, P12 and P21. Images were acquired using a 20× objective. Scale bar = 50 μm. Data are presented as mean ± SEM with *n* = 3 animals per genotype for vertical sprout analyses and *n* = 4–8 animals per genotype, depending on developmental stage, for deep plexus branching analyses. Statistical analyses for vertical sprout quantification and Dll1 expression (**e**, **f**) were performed using an unpaired two-tailed Student’s t-test, whereas branching point analyses across developmental stages (**m**) were analysed using one-way ANOVA followed by Tukey’s multiple comparison test. *p < 0.05; **p < 0.01

Given the excessive early sprouting, altered basement membrane and the ability of *Prnp*^0/0^ microglia-conditioned media to destabilize endothelial networks in vitro, we hypothesized that vascular remodelling dynamics and the assembly of the retinal vascular plexuses might be altered in the absence of PrP^C^. To test this, we quantified superficial plexus branching at P3, P5, P9, P12 and P21, covering the key developmental stages of retinal vascularization: P3-primitive vascular plexus (Fig. 5a, b); P5- stereotypical vascular plexus near optic nerve and primitive plexus in periphery (Fig. 5c, d); P9- stereotypical vascular plexus during remodelling phase (Fig. 5e, f); P12- characteristic vascular plexus in latest stages of remodelling (Fig. 6g, h); and P21, a completely remodeled vascular plexus (Fig. 5i, j). While both genotypes achieved a fully completed retinal plexus development by P21 (Fig. 5i–k), *Prnp*^0/0^ endothelium remained more ramified through P5, P9 and P12 (Fig. 5d–h, k). Notably, the pruning phase, which normally occurs between P5 and P9, was shifted to the P9 and P12 window in *Prnp*^0/0^ mice (Fig. 5f, h, k), suggesting a delayed vascular remodelling period, where the absence of PrP^C^ prevents the timely transition from an actively sprouting plexus to a stabilized, remodeled network. These findings demonstrate that PrP^C^ is required not only for initial vessel guidance, but also for the temporal transition to vascular pruning and maturation.

### Prnp^0/0^ Retina Exhibited an Increased Density of Vertical Sprouts and Vascularization During Deep Vascular Plexus Formation

This shift in vascular remodelling was further highlighted by 3D projections at P9. Vertical sprouts are crucial initiators of the deep vascular plexus, guided by tip cells [43, 44]. Using 3D confocal projections (Fig. 6a, b), we found that P9 *Prnp*^0/0^ retinas exhibited a 62% increase in vertical sprouts emerging from the superficial plexus compared to *Prnp*^+/+^ animals (Fig. 6a, b, e). Interestingly, this phenotype mirrors the effect of Delta-like ligand 1 (*Dll1)* depletion in myeloid cells [45]. Consistently, we detected a significant reduction in *Dll1* expression in *Prnp*^0/0^ retinas when compared to *Prnp*^+/+^ animals (Fig. 6f).

Since vertical sprouts drive the formation of the deeper plexuses, we next investigated deep vascularization at P9 (early) (Fig. 6g, h), P12 (intermediate) (Fig. 6i, j), and P21 (mature) (Fig. 6k, l). In *Prnp*^0/0^ mice, the deep vascular plexus was markedly more developed at P9, with an increased number of branching points (Fig. 6g, h, m) and more expanded vascular network (Fig. 6g, h). Similar to the superficial plexus, these developmental differences resolved by P21, the final stage of retina vascularization (Fig. 6k - m). These data suggest that PrP^C^ deficiency leads to an accelerated initiation of the deep vascular plexus associated with reduced *Dll1* expression.

### Laminin Deposition was Reduced in All Three Vascular Plexuses and ZO-1 Distribution was Altered in Prnp^0/0^ Retina

**Fig. 7:**
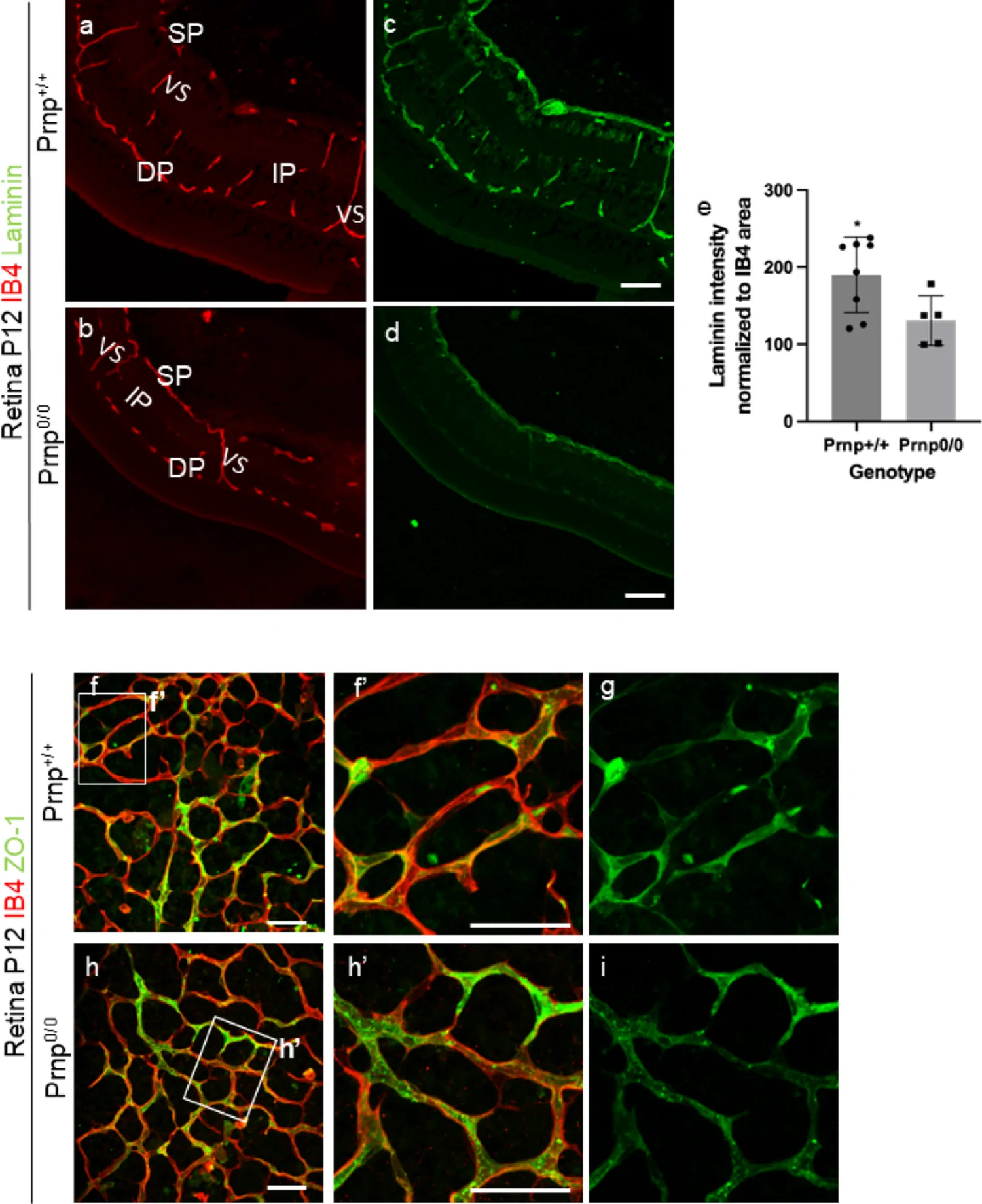
PrP^C^ regulated Laminin deposition and ZO-1 distribution in the retinal vasculature. Cryosections of postnatal day 12 (P12) mouse retinas were analysed to evaluate basement membrane components and endothelial junction organization. **a**,** b** Representative images of retinal sections from *Prnp*⁺/⁺ and *Prnp*⁰/⁰ mice stained with IB4 (red) to label blood vessels. **c**,** d** Corresponding Laminin staining (green) revealed a marked reduction in laminin deposition surrounding retinal vessels in *Prnp*⁰/⁰ mice compared with *Prnp*⁺/⁺ animals. Vascular layers are indicated as SP (superficial plexus), IP (intermediate plexus), and DP (deep plexus), with VS indicating vertical sprouts connecting the plexuses. **e** Quantification of Laminin fluorescence intensity normalized to IB4 + vascular area, showing significant reduction in *Prnp*⁰/⁰ retinas compared to *Prnp*⁺/⁺. To evaluate endothelial junction organization, whole-mount retinas were stained for IB4 (red) and ZO-1 (green) (**f**–**i**). **f’**,** h’** images amplified from **f** and **h** respectively. ZO-1 is shown qualitatively; no quantitative analysis of junctional organization was performed. Images in (**a**–**d**) were acquired using a 20× objective, whereas images in **f–i** were acquired using a 40× objective with 2.5× digital zoom. Scale bar = 50 μm. Laminin fluorescence intensity quantification (**e**) was performed using *n* = 8 *Prnp*⁺/⁺ and *n* = 6 *Prnp*⁰/⁰ animals. Qualitative analysis of ZO-1 distribution (**f**–**i**) was performed using *n* = 3 animals per genotype. Statistical analysis for Laminin fluorescence intensity was performed using an unpaired two-tailed Student’s t-test

To determine whether basement membrane defects persisted across all vascular plexuses, we performed double immunofluorescence of IB4 and Laminin in longitudinal cryosection of P12 retinas. Consistent with the qualitative observations, quantitative analysis of Laminin fluorescence normalized to IB4^+^ vascular area revealed significantly reduced laminin deposition in *Prnp*^0/0^ retinas compared to control (Fig. 7c, d, e). This density-independent analysis confirms that the reduction in Laminin is not simply due to differences in vascular coverage but reflects impaired basement membrane deposition across all vascular layers. This pan-retinal deficit reinforces our findings at P5 and suggest a generalized defect in basement membrane assembly in the absence of PrP^C^.

Because reduced Laminin can impact junctional adapter proteins like Zona Occludens-1 (ZO-1), and potentially affect blood-retinal barrier (BRB) integrity, we examined ZO-1 expression in P12 *Prnp*^+/+^ and *Prnp*^0/0^ retinas (Fig. 7e–h). While in control retinas, ZO-1 exhibited a predominantly continuous, vascular pattern (Fig. 7e, f), it appeared less uniform, with regions suggestive of discontinuity in *Prnp*^0/0^ vessels (Fig. 7g, h). These observations are consistent with a potential role for PrP^C^ in the organization of endothelial junctions. Nevertheless, further quantitative analysis will be required to confirm this effect.

### Altered Vascular Plexus Organization Accompanied by Neuroretinal Structural Remodelling in Prnp^0/0^ Retinas

**Fig. 8:**
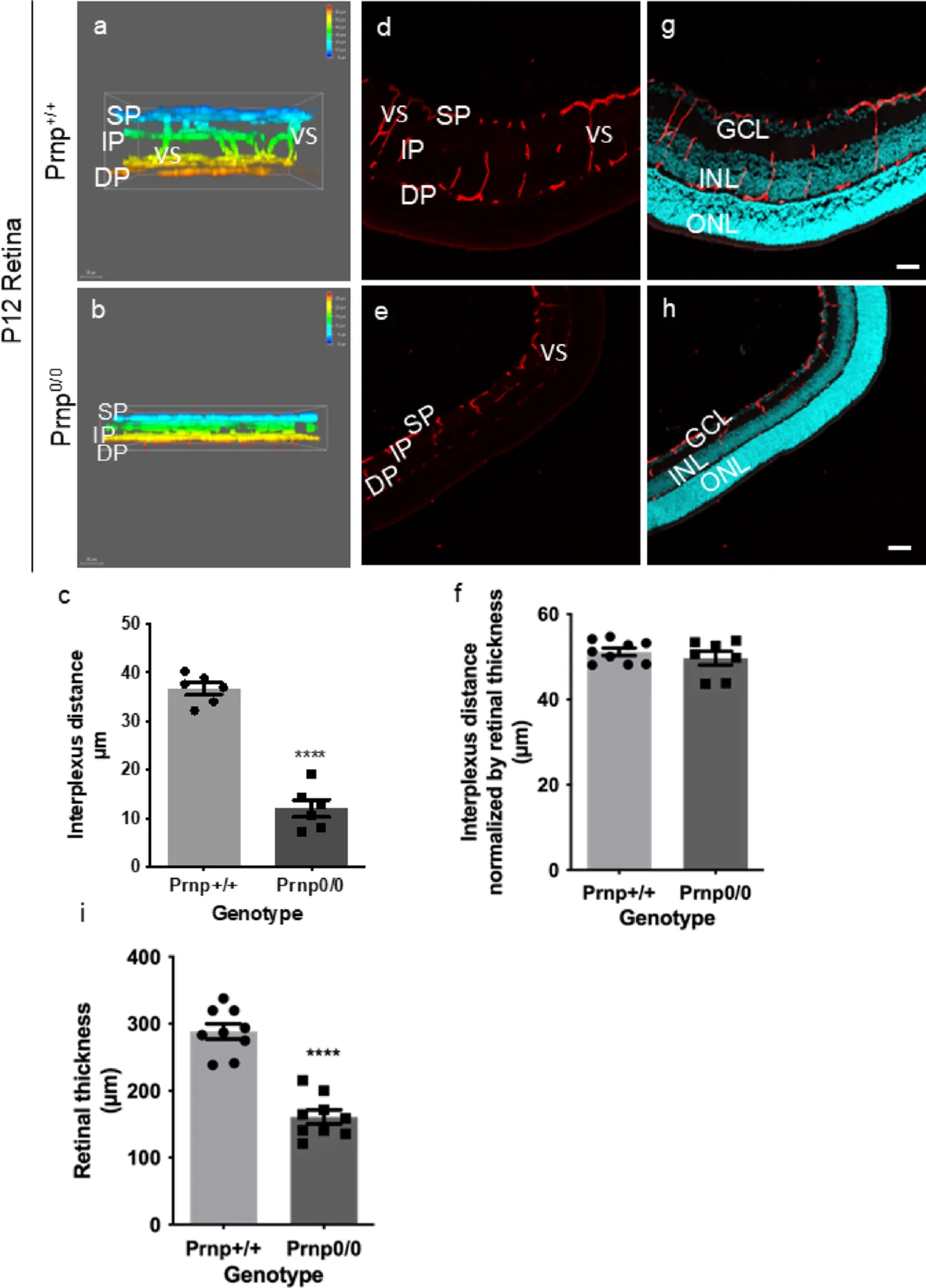
Retinal thickness and interplexus distance were reduced in the absence of PrP^C^. Three-dimensional confocal analysis of retinal vasculature was performed in P12 mouse retinas stained with isolectin B4 (IB4) to visualize vascular layers. **a**, **b** Representative 3D reconstructions of retinal vasculature from Prnp⁺/⁺ and Prnp⁰/⁰ mice showing the superficial plexus (SP), intermediate plexus (IP), and deep plexus (DP). **c** Quantification of the interplexus distance reveals a significant reduction in *Prnp*⁰/⁰ retinas compared with *Prnp*⁺/⁺ animals. To evaluate whether this difference was associated with changes in retinal morphology, cryosections of P12 retinas were stained for IB4 (red) to label blood vessels and DAPI (cyan) to visualize retinal neuronal layers. **d**, **e**, **g**, **h** Representative images of retinal cryosections from *Prnp*⁺/⁺ and *Prnp*⁰/⁰ mice showing retinal layers, including the ganglion cell layer (GCL), inner nuclear layer (INL) and outer nuclear layer (ONL). **f** Quantification of the interplexus distance normalized to retinal thickness. **i** Quantification of total retinal thickness demonstrated a significant reduction in *Prnp*⁰/⁰ retinas compared to *Prnp*^+/+^ retinas. VS vertical sprouts. Images were acquired using a 20× objective. Scale bar = 50 μm. Statistical analysis was performed using an unpaired two-tailed Student’s t-test. Data are presented as mean ± SEM with *n* = 9 animals per genotype. ****p < 0.0001

To analyse the global retinal architecture, we performed 3D projections of IB4-labeled P12 retinas (Fig. 8a, b). The inter-plexus distance was severely reduced by 67% in *Prnp*^0/0^ when compared with *Prnp*^+/+^ retinas (Fig. 8a–c), resulting in near-complete loss of vertical stratification between the three vascular layers.

This result led us to investigate the neuroretinal layer (retinal ganglion cell layer, inner nuclear layer and outer nuclear layer, GCL, INL and ONL, respectively) macroscopic organization using IB4 + labelling of vessels. While all three neuronal layers were present, they were significantly thinner and more compacted in the *Prnp*^0/0^.retina (Fig. 8a - h, f). Consequently, there was a significant reduction in global retinal thickness (Fig. 8a - h, i). These findings indicate that the absence of PrP^C^ impacts both the 3D assembly of the vascular plexuses and the overall neuroretina structural organization. Together these data suggest that PrP^C^ is critical for retinal vascular and neuronal laminar architecture.

## Discussion

In the present study, we provide evidence that the cellular prion protein (PrP^C^) contributes to the regulation of sprouting angiogenesis, modulating vascular expansion, stabilization and structural organization in the developing retina. These findings support a role for PrP^C^ as a modulator of developmental angiogenesis in the CNS.

Using *Prnp*^0/0^ and *Prnp*^+/+^ retinas, we observed increased endothelial ramification in the superficial plexus, together with a higher number of vertical sprouts and increased ramification in the deep capillary plexus. These results suggest that PrP^C^ contributes to maintaining vascular structural integrity across all retinal vascular plexuses, limiting excessive sprouting and supporting proper vessel stabilization. In contrast to previous reports of more localized vascular alterations affecting a single plexus [46–48], our data indicate a broader, pan-plexus involvement. This suggests that PrP^C^ may act as a temporal coordinator of retinal vascularization, contributing to the orderly progression of angiogenesis during postnatal retinal vascularization.

Although previous studies have linked PrP^C^ to endothelial junctional integrity [27, 49], its involvement in angiogenesis has not been clearly established. VEGF signalling represents a plausible mechanism: while VEGF-A modulates PrP^C^ expression in brain microvascular endothelial cells [27], our findings suggest a potential reciprocal interaction, in which PrP^C^ modulates glial signalling, including microglia-derived factors, during critical developmental windows [50–54]. As VEGF-A levels decline toward P21 and the BRB matures, the requirement for PrP^C^-mediated patterning may decrease, possibly contributing to the resolution of the vascular defects observed by P21. The balance between tip and stalk cells is crucial for sprouting angiogenesis. Consistent with this, *Prnp*^0/0^ retinas displayed an increased number of tip cells, together with enhanced endothelial migration in vitro, following treatment with conditioned media from *Prnp*^0/0^ microglia. These data indicate that *Prnp*^0/0^ microglia-derived factors are sufficient to influence endothelial behavior in vitro, but do not establish their necessity in vivo, nor exclude endothelial-intrinsic contributions, and an endothelial cell-autonomous role for PrP^C^ therefore remains an equally plausible contributor to the in vivo phenotype. Together these observations support the involvement of PrP^C^ in regulating tip cell phenotype and angiogenesis during CNS development, through both microglia-dependent and endothelial-intrinsic mechanisms, alongside established VEGF, Notch and TGF-β pathways [12, 55].

CNS vascular morphogenesis depends on coordinated interactions between endothelial cells and other components of the neurovascular unit, including microglia, astrocytes, pericytes and neurons [48, 56]. We observed increased microglial density in *Prnp*^0/0^ retinas accompanied by morphological changes consistent with a reactive or primed phenotype. While temporal variations in retinal microglial number are documented, the underlying mechanisms remain elusive, as cellular death appears not to be a primary contributor [57, 58]. These alterations were associated with changes in gene expression in whole-retina lysates, including *Vegf-c* levels. However, these transcriptional data reflect contributions from multiple cell types and therefore do not allow direct attribution to microglia. Nevertheless, when considered together with the observed changes in microglial distribution and morphology, as well as the functional effects of *Prnp*^*0/0*^ microglia-conditioned media on endothelial behavior in vitro, these findings support a contribution of altered neurovascular signalling to the vascular phenotype, while not allowing cell type-specific attribution.

Reduced Vegf-c expression has been associated with increased sprouting followed by impaired stabilization as described in *Vegf-c*^+/−^ models [16]. Consistent with this model, Prnp⁰/⁰ retinas showed increased endothelial branching, indicative of enhanced sprouting activity. This excessive sprouting occurs in the context of altered neurovascular signalling and impaired extracellular matrix support, as reflected by reduced laminin deposition in vivo. Supporting a potential contribution of altered microglial signalling, conditioned media from Prnp⁰/⁰ microglia was sufficient to destabilize endothelial networks in vitro. While the absolute number of empty sleeves was increased in Prnp⁰/⁰ retinas, this difference was no longer significant after normalization to vascular area, indicating that sleeve abundance largely reflects the expanded vascular network. Together, these findings support a model in which loss of PrP^C^ promotes excessive vascular expansion accompanied by alterations in vessel stabilization during retinal vascular development.

Importantly, the use of a global *Prnp* knockout model, together with gene expression analyses in whole-retina lysates, does not allow definitive resolution of cell type-specific contributions. Conditional endothelial and microglial *Prnp* knockout approaches will be required to resolve the relative contributions of each cell type. We note that, while sample sizes were in line with comparable studies, relatively small numbers of animals may limit the detection of subtle effects and contribute to variability. However, effect size analysis with 95% confidence intervals (Supplementary Fig. 2) revealed large Cohen’s d values for the primary vascular and microglial phenotypes, with confidence intervals whose lower bounds remained above the conventional threshold for a large effect (d greater than 0.8), supporting the robustness of these differences despite the relatively small sample sizes. In contrast, laminin deposition showed a more moderate effect size with a wider confidence interval (d = 1.43; 95% CI: 0.18 to 2.68), indicating a less precise estimate. A priori sample size estimates indicate that approximately nine animals per group would be required to achieve 80% power for this endpoint. Accordingly, validation in larger independent cohorts will be important to more precisely define this effect.

Beyond growth factor signalling, the vascular basement membrane is a primary determinant of vascular stability [59, 60] and tip cell behavior [41], and it is influenced by microglial interactions [48, 61]. Given the high-affinity interaction between PrP^C^ and γ1 subunit of laminin [62], we examined whether PrP^C^ loss affected basement membrane. *Prnp*^0/0^ retinas presented a marked reduction in laminin 411 and 511 isoforms, the primary vascular laminins expressed by the retinal endothelium at this stage [41], as well as a decrease in Collagen IV expression. This reduction persisted in deeper plexuses, where significantly less laminin was associated with the vasculature. These findings indicate that the absence of PrP^C^ is associated with reduced laminin deposition across all three vascular plexuses. Importantly, this reduction in laminin deposition was confirmed by quantitative analysis normalized to vascular area, indicating that it is not solely a consequence of altered vessel density but reflects a defect in basement membrane organization. This structural deficit likely contributes to the observed vascular instability, corroborating previous evidence that α4-laminin knockout mice present impaired vascular maturation [59]. Furthermore, our results are consistent with findings showing that PrP^C^ modulates laminin deposition by astrocytes and regulates neuronal differentiation in vitro [20, 23], suggesting a conserved role for the prion protein in organizing the extracellular matrix of the developing CNS. Determining whether laminin reduction reflects a direct effect of PrP^C^ loss, altered microglial signalling, or both, will require cell type-specific approaches.

Consistent with this basement membrane deficit, we observed a reduced expression of Collagen IV in *Prnp*^0/0^ retina. This likely represents a secondary consequence to the laminin reduction, as alterations in laminin expression impact the subsequent collagen IV deposition within the basal lamina [59, 63, 64].

The temporal evolution of these defects is also informative. Prnp^0/0^ animals presented excessive endothelial ramification at P5 and P12, which eventually normalized by P21 once retinal vascularization is completed. This suggests that, in the absence of PrP^C^, the retinal vasculature undergoes a delayed remodelling process. This normalization may reflect the decline in VEGF-dependent signalling as developmental angiogenesis concludes. Importantly, the increased absolute number of collagen IV^+^ empty sleeves should not be interpreted as evidence of efficient pruning at the network level. Because this difference was no longer significant after normalization to vascular area, empty sleeve abundance appears to be largely influenced by the expanded vascular network present in Prnp^0/0^ retinas. In parallel, increased early sprouting leads to higher vascular density, which may delay the apparent pruning phase and contribute to the persistence of a more complex vascular network during development. Thus, excessive vascular expansion and delayed remodelling may represent complementary outcomes of the same alteration in vascular maturation. While the exact drivers of this resolution remain to be elucidated, regulation of programmed cell death remains a candidate. PrP^C^ is well-recognized for its potent neuroprotective role [20, 23, 65] and its capacity to prevent apoptosis in various cells [66, 67]. Therefore, future studies investigating whether PrP^C^ deficiency alters apoptotic balance during vascular pruning could provide further insights into how the retinal network eventually achieves homeostatic normalization by P21.

Our results also showed that the reduction in laminin expression in Prnp^0/0^ retinas is associated with altered ZO-1 expression. This aligns with previous findings where laminin γ1 depletion led to a significant ZO-1 reduction [68]. In our dataset, qualitative assessment suggests that ZO-1 labelling in Prnp^0/0^ vessels appeared less uniform compared to controls, with regions suggestive of discontinuity. Therefore, while these data are consistent with a potential impact of PrP^C^ on the structural organization of the BRB, further quantitative and functional analyses will be required to establish this relationship.

Interestingly, when we analysed the three neuronal layers (outer nuclear, inner nuclear and ganglion cell layer), we found them to be significantly more compacted in *Prnp*^0/0^ retina. This compaction resulted in a significant reduction in overall retina thickness. Whether these structural alterations translate into functional visual deficits remains to be established; electroretinography and optokinetic tracking represent direct approaches to address this question and are a priority for future work. Given the importance of this structure for early visual processing [69], PrP^C^ may represent a regulator of coordinated neurovascular development. Future studies will determine whether the mechanisms identified here extend to pathological contexts such as ischemic retinopathies, tumour-associated vascularization, and neurodegenerative diseases.

## Supplementary Information

Below is the link to the electronic supplementary material.

Supplementary Material 2

Supplementary Material 3

## Data Availability

No datasets were generated or analysed during the current study.
